# A FAIR and open geographic data collection for the Massaciuccoli Lake basin wetland in Italy

**DOI:** 10.1016/j.dib.2025.111303

**Published:** 2025-01-16

**Authors:** Gian Luca Vannini, Pasquale Bove, Gianpaolo Coro

**Affiliations:** a Istituto di Scienza e Tecnologie dell'Informazione “A.Faedo” (ISTI), Consiglio Nazionale delle Ricerche (CNR), Via Moruzzi 1, 56124 Pisa, Italy; b Department of Agricultural, Food and Agro-Environmental Sciences (DAFE), University of Pisa, Via del Borghetto 80, 56124 Pisa, Italy; c Istituto di Geoscienze e Georisorse (IGG), Consiglio Nazionale delle Ricerche (CNR), Via Moruzzi 1, 56124 Pisa, Italy

**Keywords:** Biodiversity, Geodata, Harmonization, Environment, FAIRness, Climate change, RAMSAR, Ecosystem services

## Abstract

The creation of a catalogue of geodata harmonised over time and space is essential for describing the status of ecosystem services in wetlands. In the present work, a specific methodology has been developed for the collection and generation of spatially and temporally harmonized geographic data to describe essential ecological and socio-economic charactesristics of the Massaciuccoli Lake basin (Tuscany, Italy), while providing a re-usable methodology for other areas. We developed a methodology, which we called ‘Geodata Layers Harmonization Methodology’ (GLHM), divided into four main phases: Geodata Census (GC), Geodata Selection (GS), Geodata Alignment (GA), and Geodata Publication (GP). The first phase, GC, involved a census of geodata made available online by public institutions, prioritizing those most relevant for describing ecosystem services, such as climatic, agro-environmental, pedo-geological, and biodiversity variables, with a preference for detailed data at the local level. The metadata of the collected geodata were organized into a structured tabular format. In the GS phase, geodata were selected based on a spatial resolution compatible with regional-scale ecological models (maximum 0.0005° ≈ 50 m), and a temporal coverage that could represent from remote past to far future scenarios. Geodata with partial spatial coverage or unsuitable for ecological models were excluded. Additionally, we evaluated the compliance of the geodata published on the websites of public institutions with the Findable-Accessible-Interoperable-Reusable (FAIR) principles through a newly developed scoring system. Based on this score, we selected only the data that exceeded a minimum FAIRness threshold. In the GA phase, the selected geodata were aligned semantically (i.e., by variable meaning), temporally, and spatially. Each geodata was georeferenced using the WGS84/EPSG:4326 reference system and clipped to the boundaries of the Massaciuccoli Lake basin. Raster data were resampled to achieve a uniform spatial resolution of 0.0005°. In the last phase, GP, the aligned geodata were published on public access repositories and services: The entire collection was organized as a QGIS project with legends and a metadata table associated. An Atlas was also produced, in PDF format, which visually represented the data and metadata. The geodata and their corresponding legends were exposed through Web Map Service (WMS) and Web feature Service (WFS) standards on a GeoServer instance and catalogued in a GeoNetwork instance, compliant with the ISO19139 standard and the INSPIRE European Directive.

The collection contains 148 geo-datasets, representing 75 climatic, agro-environmental, pedo-geological, morphological, ecological, biological, and socio-economic information distributed across five temporal reference time frames: a remote past (1950–1980), a near past (1981–2015), the present (2016–2024), a near future (2025–2050), and a far future (2051–2100). Future projections are available under the Representative Concentration Pathways (RPC) 2.6, 4.5, and 8.5 to simulate low, medium, and high greenhouse gas concentration scenarios respectively.

The present geodata collection is particularly useful for wetland monitoring, management and planning. It can easily be integrated with ecological models and predictive studies to analyse the effects of climate change and anthropogenic pressures on wetlands. The GLHM methodology is applicable to other ecological contexts to create standardised structured frameworks for evaluating the status of the biodiversity and the ecosystem services and the interplay between anthropic pressures and the ecosystem response.

Specifications Table

**Table:** 

Subject	Earth and Planetary Sciences

Specific subject area	Geospatial data (geodata) on climatic, pedology, geology, morphology and biodiversity, habitat composition, phenology indices, water bodies, land use, and land cover.

Type of data	**Geodata**: 1) Raster – GeoTIFF (.tif); 2) Vector – Shapefile (.shp, .shx, .dbf, .prj);

	**Cartographic representation**: 1) Cartographic project – QGIS file (.qgz) 2) Cartographic Atlas – PDF document (.pdf) **Metadata information**: 1) Table – MS Excel (.xlsx) 2) Xml file (.xml) **Web application**: GeoServer, GeoNetwork **Data principles adopted**: Findable, Accessible, Interoperable, Reusable (FAIR).

Data collection	The data for the Massaciuccoli Lake basin study were collected following a “**Geodata Layers Harmonization Methodology**” (GLHM), constituted by four main phases: **Geodata Census (GC):** A census of geospatial data available from public institutions. **Geodata Selection (GS):** Selection of the data based on spatial and temporal characteristics and FAIR principles. Data with finer spatial resolution (maximum 0.0005°) were preferred. **Geodata Alignment (GA):** Harmonization in temporal, semantic, and spatial dimensions using GDAL and QGIS processes. **Geodata Publication (GP):** Publication of the harmonized geodata as FAIR data to an open-access repository and an INSPIRE Directive-compliant geo-catalogue.

Data source location	Each geodata in the collection was georeferenced in the WGS84/EPSG:4326 reference system. A brief general description of the area follows. City/Town/Region: Massaciuccoli Lake basin, Pisa and Lucca provinces, Tuscany. Country: Italy. Latitude and longitude bounding box for collected data: - top left corner longitude 10.22755920 latitude 43.92825171; - bottom right corner longitude 10.22755920 latitude 43.92825171.

Data accessibility	The geodata, metadata table and QGIS project are freely accessible on Zenodo. Repository name: Zenodo Data identification number: 10.5281/zenodo.10953582 Direct URL to the data: 10.5281/zenodo.10953582 The PDF document containing the ‘Massaciuccoli Lake Basin - Harmonised Geodata Atlas of Ecosystem Services’ is freely accessible on Zenodo. Repository name: Zenodo Data identification number: 10.5281/zenodo.13912261 Direct URL to data: 10.5281/zenodo.13912261 The geodata are also available via Web Map Service (WMS) and Web Features Service (WFS) standards through a GeoServer service instance, whereas metadata are accessible through a GeoNetwork service instance. These services are available in a Virtual Research Environment (VRE) of the D4Science e-Infrastructure (hosted at CNR, Pisa, Italy): Geoservice names: GeoServer v. 2.18.2/GeoNetwork v4.2.0 Service Capabilities: WMS 1.1.1, 1.3.0 - WFS 1.0.0, 1.1.0, 2.0.0 Free-subscription instructions for the D4Science VRE: Subscribe to https://itineris.d4science.org/ Request access to the ITINERIS CRITICAL ZONE VRE: https://itineris.d4science.org/group/itineris-gateway/explore Access to the GeoServer: https://itineris.d4science.org/group/itineris_criticalzonevre/geoserver Access to the GeoNetwork (use *Massaciuccoli* as a keyword to retrieve all metadata for the data collection presented in this paper): https://itineris.d4science.org/group/itineris_criticalzonevre/geonetwork

Related research article	None

## 1 Value of the Data

• **Intrinsic value of the geodata collection methodology**
  Our geodata collection derives from a census conducted among various public institutions. It includes many temporally classified variables, ensuring geographical projection and spatial resolution uniformity. We adopted a Findable, Accessible, Interoperable, Reusable (FAIR) principles and open data oriented approach for census and publication. The FAIR principles ensure that data are easy to locate (Findable), accessible through standardized methods (Accessible), compatible for integration (Interoperable), and ready for re-use in diverse contexts (Reusable). Data and metadata consultation were simplified through a structured cartographic Atlas (a PDF document) and an interactive geo-catalogue.
• **Crucial information for wetland monitoring and management**
  Our collection offers detailed, geolocalized information about the ecosystem of the Lake Massaciuccoli basin wetland, such as climatic, pedology, geology, morphology and biodiversity variables, as well as information on habitat composition, phenology indices, water bodies, land use and land cover. Conservation organisations could use the data to design habitat restoration strategies, while local governments could use them for sustainable land-use planning as an integrated part of strategic environmental assessments. This information is fundamental for developing sustainable wetland conservation and management strategies.
• **Support for ecological and predictive modelling**
  The harmonization and standardization of our geodata allow to easily integrate them with ecological models, risk assessment, and digital-twin workflows to predict how the wetland has responded and will respond to climate change and anthropogenic pressures. The data are spatially aligned (i.e., they have equal spatial resolution and projection) and are aggregated on comparable and overlapping time frames. They constitute a solid geo-information base for stakeholders interested in future changes and in mitigation and adaptation plans.
• **Re-usability of the methodology**
  The methodology we used to create our collection is easily adaptable and applicable to other ecological contexts. It can be reused to generate collections of harmonised geodata supporting comparisons, conservation planning, and ecological/ecosystem modelling. Additionally, it can be applied to already published data to check their FAIRness status and make them comply with open data principles.
• **Facilitating inter-disciplinary analyses and improving environmental policies**
  Our geodata collection can be used to explore the complex interactions between the Massaciuccoli Lake basin wetland ecosystem services and human activities in integrated frameworks combining ecology, economics and social sciences while evaluating the socio-economic impact of wetland ecosystem service changes. The information in our geodata collection can inform policymakers at local, regional, and large scales to implement effective regulations and incentives for ecosystem protection and restoration.
• **Education and Awareness-raising**
  Educational programmes and public awareness campaigns can use our geodata collection to promote greater public awareness and appreciation for the Massaciuccoli Lake basin and, consequently, encourage more sustainable behaviour among local and global communities.

## 2 Background

Wetlands are among the most important biodiversity reserves [1]. These ecosystems provide a wide range of habitats for plants, animals and aquatic organisms and play a crucial role in biological diversity conservation [2]. Wetlands act as natural filters by improving water quality and contributing to the well-being of surrounding aquatic ecosystems [3]. Climate change and the continued anthropization of the land represent a significant threat to fragile ecosystems, such as wetlands, which have suffered a significant reduction in recent decades [4], with a decrease of 70 % during the 20th century globally [5]. This decrease has had a negative impact on the biodiversity and key ecosystem services provided by these areas, such as water quality, food supply, carbon sequestration, and social and tourism aspects [6,7]. Many public institutions acquire cartographic information related to these areas according to their expertise and scopes. However, geodata can be duplicated across these institutions and might have different representation scales and reference time frames. This scenario calls for creating a harmonised collection of geospatial data that would provide a spatially and temporally detailed knowledge for wetland ecosystem services.

## 3 Data Description

The present paper describes a harmonized and standardized geodata collection containing 148 datasets representing 75 climatic, agro-environmental, pedo-geological, morphological, ecological, biological, and socio-economic variables related to the wetland area of the Massaciuccoli Lake basin (Tuscany, Italy). Each geodata file contains a single variable. The geodata, and thus each variable, are associated with five time frames: a remote past (1950–1980); a near past (1981–2015); the present (2016–2024); a near future (2025–2050); a far feature (2051–2100). Furthermore, the geodata associated with the near future and far future frames are distinguished over different greenhouse gas concentration scenarios based on the Representative Concentration Pathways (RCP) 2.6 (low concentration), 4.5 (medium concentration), and 8.5 (high concentration). Our geodata collection contains 109 raster files (in the GeoTIFF format) and 39 vector files (in the Shapefile format), all projected in the WGS84/ EPSG:4326 reference system.

**Fig. 1:**
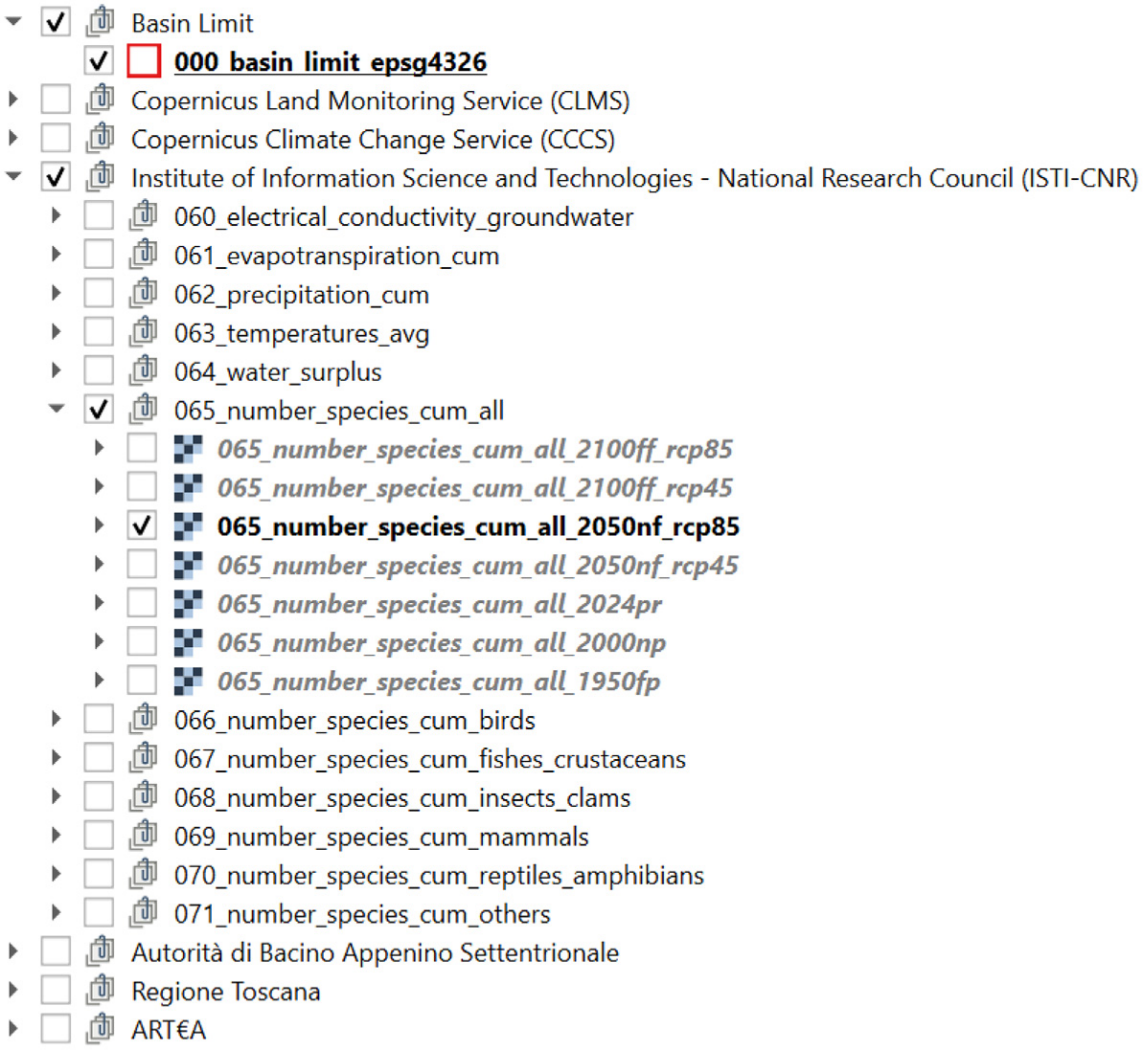
Organization of the harmonized geodata collection in the QGIS table of content.

The Raster geodata were resampled to a spatial resolution of 0.0005° (≈ 50 m), which is compatible with regional-scale ecological models. All data were aligned and clipped to the boundaries of the Massaciuccoli Lake basin. All harmonized geodata were packaged into a QGIS-software project file (‘Massaciuccoli_selected_data.qgz’), along with their corresponding legends and visualization styles. The QGIS project groups the geodata according to their primary sources (i.e., the public institutions that originally released the data) and then by the standard names of the variables, expressed in compliance with the Climate and Forecast (CF) conventions [8] with the addition of customized reference codes (Fig. 1). The standard name is indeed composed of (i) the dataset *id* from the metadata table (Table 1), (ii) a CF-compliant *geodata name*, (iii) the reference *time frame*, and, when available, (iv) the reference *RCP scenario*.

**Table 1:** List of harmonized geodata with ’Id’, measurement ‘Unit’ and ‘Chronological proximity and potential aggregation’.

For example, ‘063_temperatures_avg_2100ff_rcp85’ is structured as ‘id’= ‘063’, ‘geodata name’= ‘temperatures_avg’, ‘time period’= ‘2100ff’, and ‘RCP scenario’= ‘rcp85’. The QGIS project includes a folder (‘massaciuccoli_selected_data’) that contains all the files of the data collection. These files are organized into subfolders by ‘id’ and ‘geodata name’, which reflects the organization shown in the QGIS project. For instance, the subfolder ‘063_temperatures_avg’ contains all geodata files related to the variable ‘Temperatures average’ (063_temperatures_avg_1950fp, 063_temperatures_avg_2000np, 063_temperatures_avg_2024pr, 063_temperatures_avg_2050ff_rcp45, 063_temperatures_avg_2050ff_rcp85, 063_temperatures_avg_2100ff_rcp45, 063_temperatures_avg_2100ff_rcp85). The legends describing the cartographic representation have been kept in the original language of the original geodata.

The ‘Massaciuccoli_lake_basin_metadata.xlsx’ file (in MS Excel format) contains the metadata table (Table 2). It also includes the metadata of the geodata discarded during the selection phase due to insufficient quality, relevance, or FAIRness levels (as explained in ‘Experimental design, materials and methods’). By default, these data are hidden through de-activable column filters. Each table reports the following information for each geodata: the name, the description, the original spatial features, the primary source from which it was acquired, and the FAIRness level. An additional sheet (‘Legend’) describes the symbology used to represent the data content.

**Table 2:** Description of metadata table fields contained in the ‘Massaciuccoli_lake_basin _metadata.xlsx’ file, ‘Metadata and selection’ sheet.

**Fig. 2:**
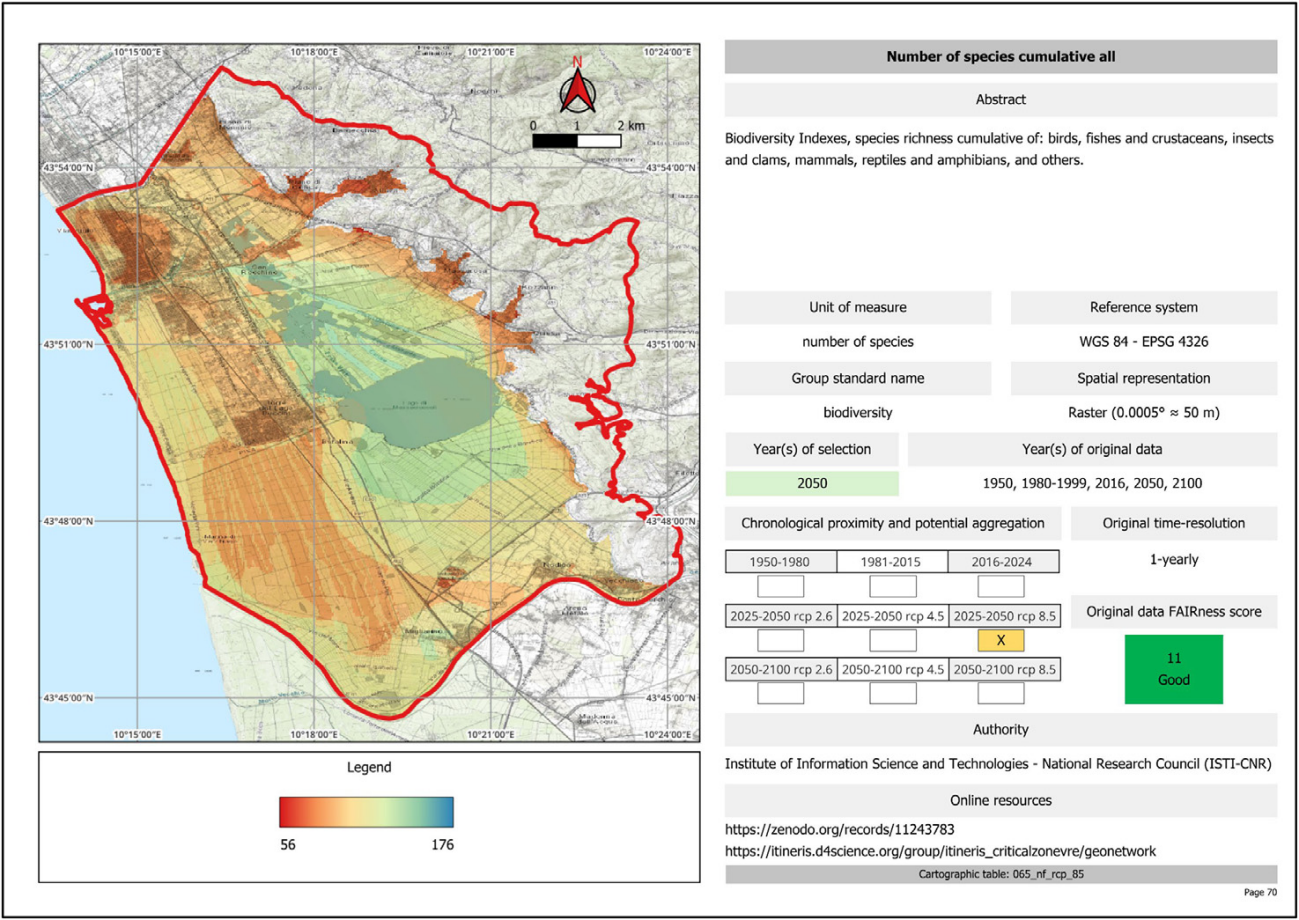
Example of cartographic table in our Atlas.

As an additional representation, we produced a PDF document containing the Cartographic Atlas (‘Massaciuccoli Lake Basin - Harmonized Geodata Atlas of Ecosystem Services’, Massaciuccoli_lake_basin_harmonized_geodata_atlas.pdf). This representation facilitates the consultation of the geodata collection. The document includes two table indexes: ‘Table and index of cartographic coverages’, located at the beginning of the Atlas, which provides a summary of the metadata for all harmonised geodata, and ‘Analytical index’, located at the end, which allows users to find the cartographic table of a geodata quickly. The cartographic table is a graphic representation of the data with the corresponding legend on the left, and a structured description (metadata) of the geodata on the right (Fig. 2). The reference time frame is highlighted in orange to give a general sense of data change over time while proceeding through the pages. Indeed, the tables of one parameter over different time frames are located on consecutive pages.

**Fig. 3:**
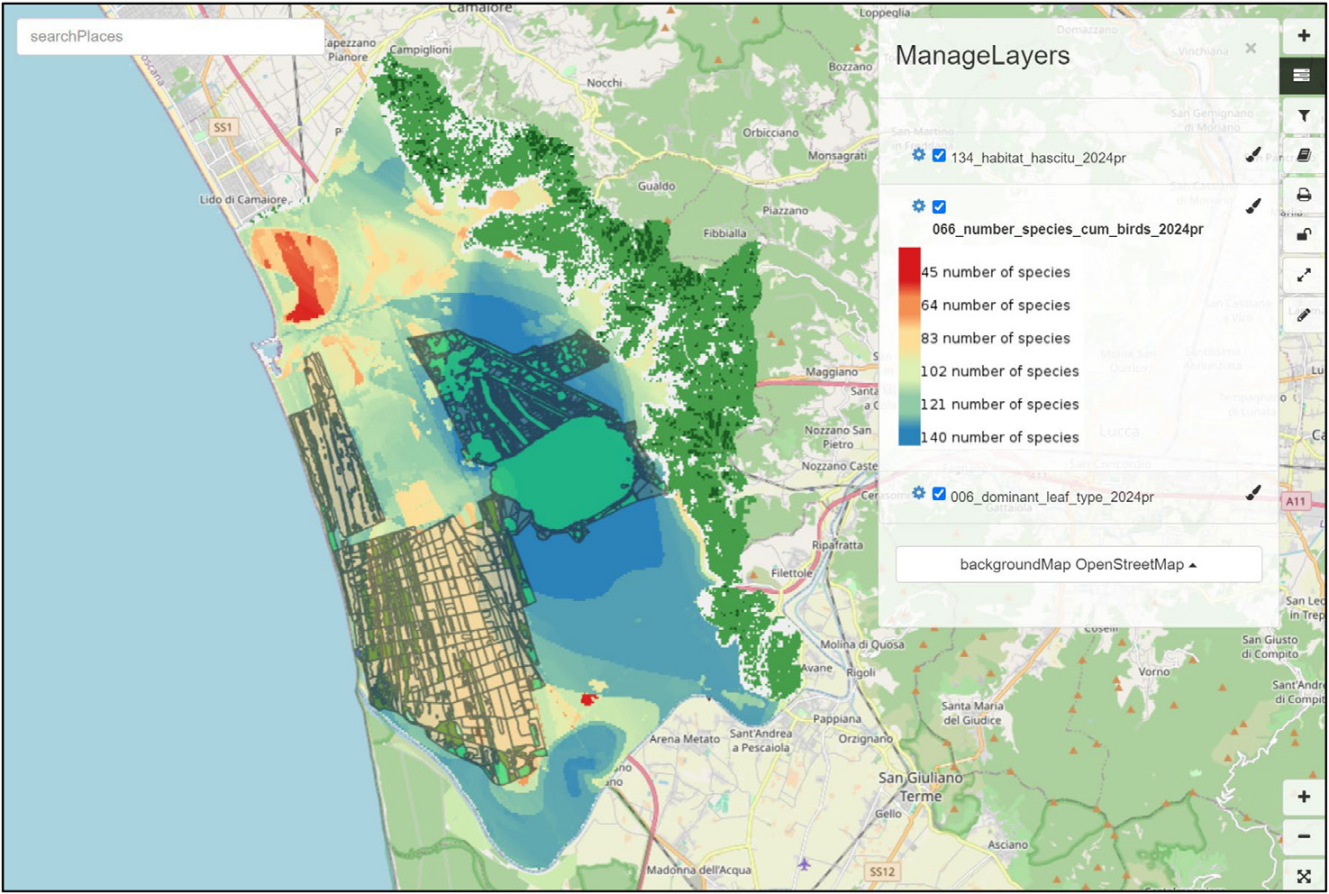
Example of a custom map created on the GeoNetwork service instance, overlaying three datasets: Dominant leaf type (present, 2016–2024), Number of species cumulative birds (present, 2016–2024), and Habitat-HASCITu (present, 2016–2024).

Finally, the GeoServer and GeoNetwork service instances of the ITINERIS Critical Zone Virtual Research Environment (hosted by the D4Science e-infrastructure [9]) allow users to interactively inspect and download maps, data, and metadata directly from a web browser. An integrated online visualiser in GeoNetwork allows users to create custom maps by overlaying multiple geodata layers without needing desktop software (Fig. 3).

## 4 Experimental Design, Materials and Methods

We designed the geodata harmonization methodology with the aim of obtaining a reliable, unique, homogeneous, geo-localised data collection, which was spatially and temporally aligned. We applied our methodology to the wetland area of the Massaciuccoli Lake basin in Tuscany, Italy. We named our general methodology “**Geodata Layers Harmonization Methodology**” (GLHM). It is divided into four main phases: **Geodata Census**, in which we compile a census of the geodata available from public institutions; **Geodata Selection**, based on the census, in which we select the geodata according to their geospatial and temporal characteristics and their usefulness for ecosystem and spatial planning models. In this phase we also use an automatic process to check for the possibility of directly downloading each geodata from the primary source and assess their compliance with FAIR principles; **Geodata Alignment**, in which the geodata are semi-automatically aligned semantically, temporally, and spatially to ensure data consistency and homogeneity; **Geodata Publication**, in which the geodata and their related metadata are semi-automatically made compliant with FAIR principles and accessible through an open-access repository, an interactive geo-catalogue and a geo-service. In the following, we explain each phase of our GLHM.

### 4.1 Geodata layers harmonization methodology

In the **Geodata Census (GC)** phase, the information on the geodata is collected and organized in a tabular format. A screening is conducted to identify the geodata that might be useful for describing or understanding the ecosystem services of the wetland. This information is indeed crucial for ecosystem modellers and spatial planners, who are our target stakeholders. The GC phase assigns a higher priority to collecting data from public institutions that work on a local scale. These institutions typically collect more detailed geodata than those working at larger scales (e.g., regional, national, or European). For example, the geological data collected by the Autorità di Bacino Distrettuale dell'Appennino Settentrionale (a sub-regional monitoring authority) are generally more detailed than those from the Regione Toscana (the Tuscany region monitoring authority). Consequently, when different geodata describe the same variable, those belonging to more localized authorities are preferred and reported instead of the others. This operation avoids repeating the same information in our catalogue while providing more specialized and reliable information.

As for the Massaciuccoli Lake basin data, the GC phase identified 151 geo-variables comprising 783 geodata. The GC process can be summarised as follows:

**Geodata Census process:**

*Identification*

• Identify the geodata distributed by the main public institutions providing climatic, ecological, environmental, pedological, geological, morphological, agronomic, anthropic, biodiversity, and habitat information on the wetland area of interest (the Massaciuccoli Lake basin in the case study of the present paper).

*Reporting*

• Report the geospatial, temporal, descriptive, and origin information of the resource, i.e., the original geodata name, the public institution that produced it, the geodata description, the unit of measure, the spatial projection, coverage, representation (raster/vector), and resolution, the time coverage and resolution, and the primary source used to access the geodata.

The **Geodata Selection (GS)** phase uses a 0.0005° (∼50 m) spatial resolution threshold to select the geodata that can proceed to the subsequent phases. This threshold aligns with other studies [10,11] assessing its sufficiency for regional-scale ecological models and monitoring analyses. A coarser resolution (e.g., 0.0010°, ∼100 m) would not have been adequate to describe the territorial variables contained in the geodata accurately. On the other hand, a finer resolution would have discarded too much data without adding benefits for our target stakeholders. An exception is made for the geodata of projections in the future, which are retained even at coarser resolutions when they corresponded to a variable also available at a finer resolution. In fact, these data are useful for projecting higher resolution geodata over time [11]. Among the geodata with sufficient spatial resolution and describing the same variable over comparable periods, we retain those presenting the finest temporal resolution (e.g., monthly data instead of annual over the same period or annual instead of decadal). Geodata that only partially cover the study area (e.g., surveys conducted at a sub-area scale) and those not useful for ecosystem models and spatial planning (e.g., sub-area territorial division lines) are also excluded.

In the GS phase, we further select the data based on the compliance of the geodata with the FAIR principles [12]. For this purpose, we developed a FAIRness score that quantifies how much a geodata complies with the FAIR principles (explained in the methodology reported below). This score is automatically calculated through a batch process by the Microsoft Copilot chatbot based on a prompt embedding the rules of our FAIRness scoring and classification algorithm described below. As for the Massaciuccoli Lake data, the scores were hand-revised to check for their accuracy and consistency. We agreed with ∼80% of the assessments.

The GS phase analysed geo-variables and geodata identified in GC phase, and finally selected 75 geo-variables comprising 148 geodata (including the geodata of the boundaries of the Massaciuccoli Lake basin). The GS process can be summarised as follows:

**Geodata Selection process**

*Spatial selection*

• Acquisition of geodata with ground resolution higher than or equal to a fixed threshold (e.g., 0.0005°);
• Selection of future projections of these data, even at a coarser resolution;
• Selection of geodata with complete (not partial) spatial coverage.

*Temporal selection*

• When different geodata describe the same variable, select those covering a longer temporal span with a finer time resolution.

*Usage selection*

• Acquire only geodata useful for ecosystem modelling and spatial planning.

*Compliance with FAIR principles and direct download selection*

• For each subheading of the FAIR principles (Findable F1, F2, F3, F4; Accessible A1, A1.1, A1.2, A2; Interoperable I1, I2, I3; Reusable R1, R1.1, R1.2, R1.3 – as described in Table 2) [12], assign the following scores:

**Table:** 

**Subheading score**	
Score	Description

0	Geodata and metadata do not met the requirements specified by subheading

0.5	Geodata and metadata partially met the requirements specified by the subheading

1	Geodata and metadata fully met the requirements specified by subheading

• Create a FAIRness index for each geodata as follows (using the Copilot chatbot to assess the scores):

° Sum the scores of each individual subheading of the FAIR principles, as shown in the following example for the “Corine Land Cover (CLC) backbone” (id: 1) geodata:

**Table:** 

**FAIRness features**															
Findable				Accessible				Interoperable			Reusable				Total score
F1	F2	F3	F4	A1	A1.1	A1.2	A2	I1	I2	I3	R1	R1.1	R1.2	R1.3	
1	1	1	1	1	1	1	1	1	0.5	1	1	1	1	1	14.5

° Classify the geodata FAIRness based on the following values of the FAIRness index:

**Table:** 

**Total Score**			
From	To	FAIRness grade	
0	6		Insufficient

6.5	10.5		Sufficient

11	15		Good

° Revise the classification by checking that the F1, F2, F3, and F4 subheadings have a minimum score ≥ 0.5 because they are essential components of the harmonization process. If this condition is unsatisfied, reclassify the geodata as *Insufficient*;

° Revise the classification based on the direct accessibility of the geodata for download. If the data cannot be downloaded, then classify the geodata as *Insufficient*;

° Discard all *Insufficient* geodata and pass the other data to the next harmonization steps.

In the **alignment phase (GA)**, each geodata selected in the previous phase (GS) is assigned to a reference time frame and a year considered the most representative of the time frame. Five distinct time frames were defined for the Massaciuccoli Lake basin: a ‘remote past’ (1950–1980, representative year=1950), a ‘near past’ (1981–2015, representative year=2000), the ‘present’ (2016–2024, representative year=2024), a ‘near future’ (2025–2050, representative year=2050), and a ‘far future’ (2051–2100, representative year=2100), in line with previous studies [11]. Future projections were simulated through to Representative Concentration Pathways (RCPs) 2.6 (low greenhouse gas concentration), 4.5 (average greenhouse gas concentration) and 8.5 (high greenhouse gas concentration).

The GA phase disambiguates different geodata referring to the same variable and time frame by retaining those closest to the representative year of the aggregation time frame and possibly defined over multiple years. For example, between two datasets describing ‘Land use and land cover’ (id: 15 and 146) for the ‘present’ time frame (2016–2024), one for 2018 and another for 2019, the GA phase selected the one of 2019 because it was closer to 2024 (the representative year). Another example was the dataset of ‘Cumulative Precipitation’, identified with id 56, 62, 49 and 148, among which the dataset with id 62 was selected because, differently from the others, it covered all five time frames compared to the others. In the GA phase, equivalence cases are further resolved by retaining the geodata with the highest FAIRness score. For instance, with regard to the variable ‘Water surplus’, the dataset with id 64 had a higher FAIRness index than the dataset with id 81 (11 versus 6.5, respectively).

In the GA phase, GDAL and QGIS batch processing workflows are used to reproject both raster and vector geodata onto the same reference system (WGS84/EPSG:4326) and clip them to boundaries of the study area. This process involves annual time-averaging of the variables, while ensuring consistency and comparability across the datasets within each temporal frame. Additionally, raster data are resampled to a standardized spatial resolution (0.0005° for the Massaciuccoli basin).

In the GA phase, all Massaciuccoli Lake basin geodata selected in the GS phase were spatially and temporally aligned (75 geo-variables comprising 148 geodata). The GA process can be summarised as follows:

**Geodata Alignment process:**

*Temporal aggregation*

• Assign the selected geodata to one among the following time frames: 1) Remote past; 2) Near past; 3) Present; 4) Near future; 5) Far future. Use multiple future scenarios (e.g., the RCP scenarios) if available.

*Semantic alignment*

• Assign a standard name to each geodata in order to identify the same variable;
• If two or more geodata refer to the same time frame and represent the same variable (and therefore have the same standard name), select: 1) the one closest in time to the representative year; otherwise, 2) the one with the highest FAIRness index; otherwise, 3) the one with the longest time span.

**Spatial alignment**

• Georeference the data in the WGS84/EPSG:4326 reference system;
• Crop the geodata on the boundaries of the survey area (i.e., the Massacciuccoli Lake basin in our case study);
• Average the geodata annually if the time resolution is lower (e.g., monthly or daily);
• Resample the raster geodata to the target spatial resolution (e.g., 0.0005°).

In the **Geodata Publication (GP)** phase, the set of geodata selected and aligned by the previous phases is organized into a QGIS project. This (manual) operation enriches the data with legends and visualization styles and eventually packs the project into a ZIP file. Additionally, this phase produces a table containing the metadata for each geodata layer in MS Excel format.

The QGIS project and the metadata table are used to produce a Cartographic Atlas as a PDF document to show the data and their evolution over time to a wider audience. The geodata and legends are imported into a GeoServer instance - hosted in the D4Science ITINERIS CRITICAL ZONE Virtual Research Environment in our case study - via the GeoServer API through shell scripts. GeoServer enables standardized Open Geospatial Consortium (OGC) service descriptions for the imported data to enhance interoperability with visualization and analytics software. These descriptions are embedded in XML metadata documents that are compliant with the ISO 19139 standard and the European INSPIRE Directive [13]. The XML documents are automatically built and imported through shell scripts onto a GeoNetwork service instance (still hosted by D4science) through the GeoNetwork API. The GeoNetwork catalogue is a structured and machine-accessible representation of our harmonised and aligned geodata collection.

In the GP phase, all Massaciuccoli Lake basin geodata aligned during the GA phase were published (75 geo-variables comprising 148 geodata). The GP process can be summarised as follows:

**Geodata Publication process:**

*QGIS project*

• Organize the geodata into a cartographic project (QGIS file) and a metadata table;
• Publish this information onto an Open Science oriented platform (D4Science) as openly accessible data.

*Atlas*

• Create a Cartographic Atlas in PDF format (printable in hard copy), containing a graphical representation of the data and metadata;
• Publish this document as an openly accessible cartography onto D4Science.

*GeoServer/GeoNetwork*

• Import the geodata into a GeoServer service to expose them through OGC standards (Web Map Service, Web Coverage Service, and Web Feature Service);
• Import the metadata into a GeoNetwork service instance, exposing metadata in the ISO19139 standard, compliant with the INSPIRE Directive [13].

As for the Massaciuccoli Lake basin, all 148 harmonised geodata layers were made openly available on a Zenodo-hosted repository and within the ITINERIS Virtual Research Environment, as reported in the Specifications Table.

## Limitations

The present geodata collection is released under the ‘Attribution 4.0 International’ (CC-BY 4.0) licence.

Further specifications and details on geodata acquisition and restitution techniques and technologies are available on the websites of the public institutions that produced the original geodata. The ‘primary source’ column of the ‘Metadata and selection’ sheet of the ‘Massaciuccoli_lake_basin_metadata.xlxs’ table reports the URLs of the web pages of the original geodata.

## Ethics Statement

The authors confirm that they have read the ethical requirements for publication in Data in Brief and confirm that the current work does not involve human subjects, animal experiments, or any data collected from social media platforms.

As the geodata contained in the dataset are already part of the public domain under CC-BY Licence, no permission to use primary data was needed.

## Credit Author Statement

**Gian Luca Vannini:** Conceptualization, Methodology, Software, Validation, Formal analysis, Investigation, Data curation, Writing – original draft, Visualization, Writing – review & editing. **Pasquale Bove:** Validation. **Gianpaolo Coro:** Conceptualization, Software, Writing – review & editing, Supervision, Funding acquisition.

## Data Availability

ZenodoMassaciuccoli Lake Basin - Harmonized Geographic Atlas for Ecosystem Services Assessment (Original data). ZenodoMassaciuccoli Lake basin in Tuscany, Italy. Datasets of 75 environmental, geomorphologic, and socio-economic variables associated from remote past to remote future (Original data).
